# Construction of a high-density genetic map for sesame based on large scale marker development by specific length amplified fragment (SLAF) sequencing

**DOI:** 10.1186/1471-2229-13-141

**Published:** 2013-09-24

**Authors:** Yanxin Zhang, Linhai Wang, Huaigen Xin, Donghua Li, Chouxian Ma, Xia Ding, Weiguo Hong, Xiurong Zhang

**Affiliations:** 1 Oil Crops Research Institute of the Chinese Academy of Agricultural Sciences, Key Laboratory of Biology and Genetic Improvement of Oil Crops, Ministry of Agriculture, No.2 Xudong 2nd Rd, 430062 Wuhan, China; 2 Biomarker Technologies Corporation, 101300 Beijing, China

**Keywords:** *Sesamum indicum* L, High-density, Genetic map, Linkage analysis, Sequencing

## Abstract

**Background:**

The genetics and molecular biology of sesame has only recently begun to be studied even though sesame is an important oil seed crop. A high-density genetic map for sesame has not been published yet due to a lack of sufficient molecular markers. Specific length amplified fragment sequencing (SLAF-seq) is a recently developed high-resolution strategy for large-scale *de novo* SNP discovery and genotyping. SLAF-seq was employed in this study to obtain sufficient markers to construct a high-density genetic map for sesame.

**Results:**

In total, 28.21 Gb of data containing 201,488,285 pair-end reads was obtained after sequencing. The average coverage for each SLAF marker was 23.48-fold in the male parent, 23.38-fold in the female parent, and 14.46-fold average in each F_2_ individual. In total, 71,793 high-quality SLAFs were detected of which 3,673 SLAFs were polymorphic and 1,272 of the polymorphic markers met the requirements for use in the construction of a genetic map. The final map included 1,233 markers on the 15 linkage groups (LGs) and was 1,474.87 cM in length with an average distance of 1.20 cM between adjacent markers. To our knowledge, this map is the densest genetic linkage map to date for sesame. 'SNP_only’ markers accounted for 87.51% of the markers on the map. A total of 205 markers on the map showed significant (*P* < 0.05) segregation distortion.

**Conclusions:**

We report here the first high-density genetic map for sesame. The map was constructed using an F_2_ population and the SLAF-seq approach, which allowed the efficient development of a large number of polymorphic markers in a short time. Results of this study will not only provide a platform for gene/QTL fine mapping, map-based gene isolation, and molecular breeding for sesame, but will also serve as a reference for positioning sequence scaffolds on a physical map, to assist in the process of assembling the sesame genome sequence.

## Background

*Sesamum indicum* L. (sesame), a diploid species (2n = 26), is one of the most ancient oil seed crops [1]. About 4 million tons of sesame seeds are produced each year worldwide (http://faostat.fao.org/). Sesame seeds are an important source of high-quality oil containing natural antioxidants such as sesamin and sesamol. Despite its economic importance and nutritional value, the genetic and molecular biology study of sesame began only recently. Just one genetic map has been published for sesame [2] and quantitative trail loci (QTL) mapping has not been reported.

A genetic map, especially a high-density genetic map, provides an important foundation for QTL mapping [3-5] and for anchoring sequence scaffolds [6,7]. Large-scale microsatellite marker development is an important approach to obtain sufficient numbers of markers to construct a high-density genetic map. This approach was used for tobacco (*Nicotiana tabacum* L.) [8] and Japanese flounder (*Paralichthys olivaceus*) [5], constructing high-density genetic map with an average interval of less than 1.5 cM between markers, and the developed SSR markers will also be important tools to further advance genome analysis. Another approach to construct a high-density genetic map is to utilize several types of available markers in a single study. Troggio et al. [9] published a dense genetic linkage map for grapevine that was constructed using 483 single-nucleotide polymorphism (SNP)-based genetic markers, 132 simple sequence repeats (SSRs), and 379 amplified fragment length polymorphisms (AFLPs). The SNP-based markers were developed from BAC-end sequence resources, and their inclusion significantly increased the density of the map. Wang et al. [10] constructed a high-density genetic linkage map for cabbage with 602 SSRs and 625 SNPs that spanned a total of 1,197.9 cM with an average of 0.98 cM between adjacent loci. In this case, the SNP markers were developed by resequencing the other parent of a mapping population.

SNPs are more useful as genetic markers than many conventional markers because they are the most abundant and stabile form of genetic variation in most genomes [11]. Sequence polymorphisms introduced by SNPs are easy to compile in a database and are useful for evolutionary studies [9]. High-throughput methods for SNP identification and genotyping have been rapidly developed to exploit these advantages. Miller et al. [12] developed a rapid and cost-effective polymorphism identification and genotyping approach using restriction site-associated DNA (RAD) markers. This approach was used for rapid SNP discovery and genetic linkage mapping in many organisms including stickleback, *Neurospora crassa*, *Plutella xylostella*, barley, and grape [13-16]. Peterson et al. [17] reported a modified low-cost RAD sequencing (RADseq) approach called double digest RADseq as a complete laboratory protocol requiring no prior genomic knowledge. Poland et al. [18] also developed a novel two-enzyme genotyping-by-sequencing (GBS) approach and used it to construct high-density genetic maps for barley and wheat. The use of two enzymes differed from the original GBS protocol in that amplified fragments in the two-enzyme libraries all consisted of the barcoded forward adapter and the common reverse adapter. This method of library construction greatly simplified the quantification of the library prior to sequencing. Recently, Sun et al. [19] reported the development of specific length amplified fragment sequencing (SLAF**-**seq) as a high-resolution strategy for large-scale *de novo* SNP discovery and genotyping. The efficiency of this approach was tested on data from rice and soybean and used to create a genetic map for common carp (*Cyprinus carpio* L.), which was the highest-density genetic map yet for any organism without the benefit of a reference genome sequence.

Since 1999, several kinds of markers have been used to assess the genetic diversity of sesame accessions including random amplified polymorphic DNA (RAPD) [20,21], inter-simple sequence repeat (ISSR) [22,23], AFLP [24,25], sequence-related amplified polymorphism (SRAP) [26,27], and random selective amplification of microsatellite polymorphic loci (RSAMPL) [2], but no SSR markers were available for sesame until 2005. Dixit et al. [28] developed the first series of SSR markers for sesame and more than 500 SSR markers have been developed and published to date [2,28-31]. Despite the demonstrated importance of SSR markers for constructing genetic maps in many crops, the quantity of available sesame SSR markers was not sufficient to meet the requirements of genetic map construction due to the low polymorphism rate in these markers. Wei et al. [2] constructed the first genetic map for sesame using 120 EST-SSRs, 256 AFLPs, and 8,576 RSAMPLs, but only 220 of these markers (8 EST-SSRs, 25 AFLPs, and 187 RSAMPLs) were mapped in 30 linkage groups covering 936.72 cM with an average marker spacing of 4.93 cM. Wei et al. [2] pointed out the need for additional markers to obtain an accurate length of the sesame genome and high-density map coverage. Subsequently, Zhang et al. [30] integrated 14 genic SSRs into 9 main linkage groups on this map. But a high-density genetic map for sesame has not been published yet due to a lack of sufficient molecular markers.

In this study, we employed the recently developed SLAF-seq approach to achieve the first rapid mass discovery of SNP and insertion-deletion (InDel) markers for sesame. Using these newly developed markers, a high-density genetic map was constructed and its characteristics were investigated, the method used in this study for developing markers and future applications for this genetic map were also discussed.

## Results

### Analysis of SLAF-seq data and SLAF markers

After SLAF library construction and high-throughput sequencing, 28.21 Gb of data containing 201,488,285 pair-end reads was obtained with each read being ~70 bp in length. The Q20 (means a quality score of 20, indicating a 1% chance of an error, and thus 99% confidence) ratio was 74.32% and guanine-cytosine (GC) content was 40.60%. Of these high-quality data, ~158 Mb were from the male parent with 2,259,333 reads and ~157 Mb were from the female parent with 2,246,084 reads. Read numbers for the 107 individuals in the F_2_ population ranged from 824,263 to 3,122,925 with an average of 1,416,287.

**Figure 1:**
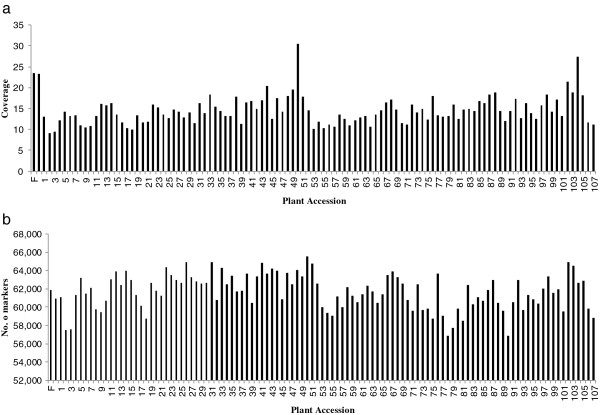
**Coverage and number of markers for each of the F**_**2 **_**individual and their parents.** The *x*-axes in **a** and **b** indicate the plant accession including the female parent and the male parent followed by each of the F_2_ individuals, the y-axes indicates coverage in a and number of markers in b.

The numbers of SLAFs in the male and female parents were 61,909 and 60,949, respectively. The read numbers for SLAFs were 1,453,579 and 1,425,202 in the male and female parents, respectively. The average coverage for each marker was 23.48-fold in the male parent and 23.38-fold in the female parent. In the F_2_ population, the numbers of SLAF markers in each individual ranged from 56,852 to 65,545 with an average of 61,705. The read numbers for SLAFs ranged from 527,734 to 2,000,299 with an average of 898,509, and the coverage ranged from 9.18-fold to 30.52-fold with an average of 14.46-fold (Figure 1).

**Figure 2:**
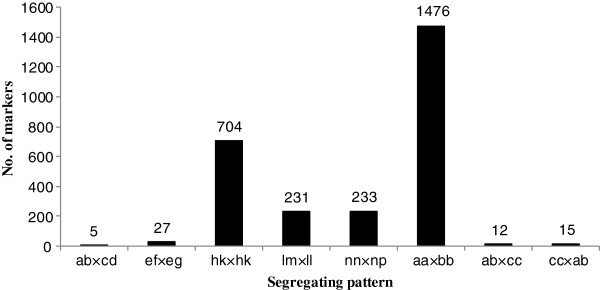
Number of markers for eight segregation patterns.

Among the 71,793 high-quality SLAFs that were detected, 3,673 were polymorphic with a polymorphism rate of only 5.12% (Table 1). Of the 3,673 polymorphic SLAFs, 2,703 were classified into eight segregation patterns (Figure 2). An F_2_ population is obtained by selfing the F_1_ of a cross between two fully homozygous parents with genotype aa or bb. Therefore, only the aa × bb segregation pattern in the F_2_ population was used to construct a genetic map, and 1,476 markers fell into this class. Among these 1,476 markers, 1,272 markers had more than 20-fold of parental sequence depth, and more than 14-fold of individual sequence depth, and over 80% integrity of SLAF tags, and these were used for the genetic map construction.

**Table 1:** SLAF markers mining results

	**Number of SLAF markers**	**Number of reads**	**Ratio**
Polymorphisms	3,673	5,779,302	5.12%
Non-polymorphisms	68,120	92,992,719	94.88%
Total	71,793	98,772,021	100.00%

### Basic characteristics of the genetic map

After linkage analysis, 1,233 (Additional file 1) of the 1,272 (Additional file 2) markers were mapped onto the genetic map, while other 39 markers were failed to be linked to any group. Coverage of these markers was 23.83-fold in the male parent, 23.32-fold in the female parent, and 14.96-fold in each F_2_ individual on average. The integrity of each marker among the 107 F_2_ individuals was also a key parameter for controlling map quality. All of the markers on the map had 98% integrity on average.

**Figure 3:**
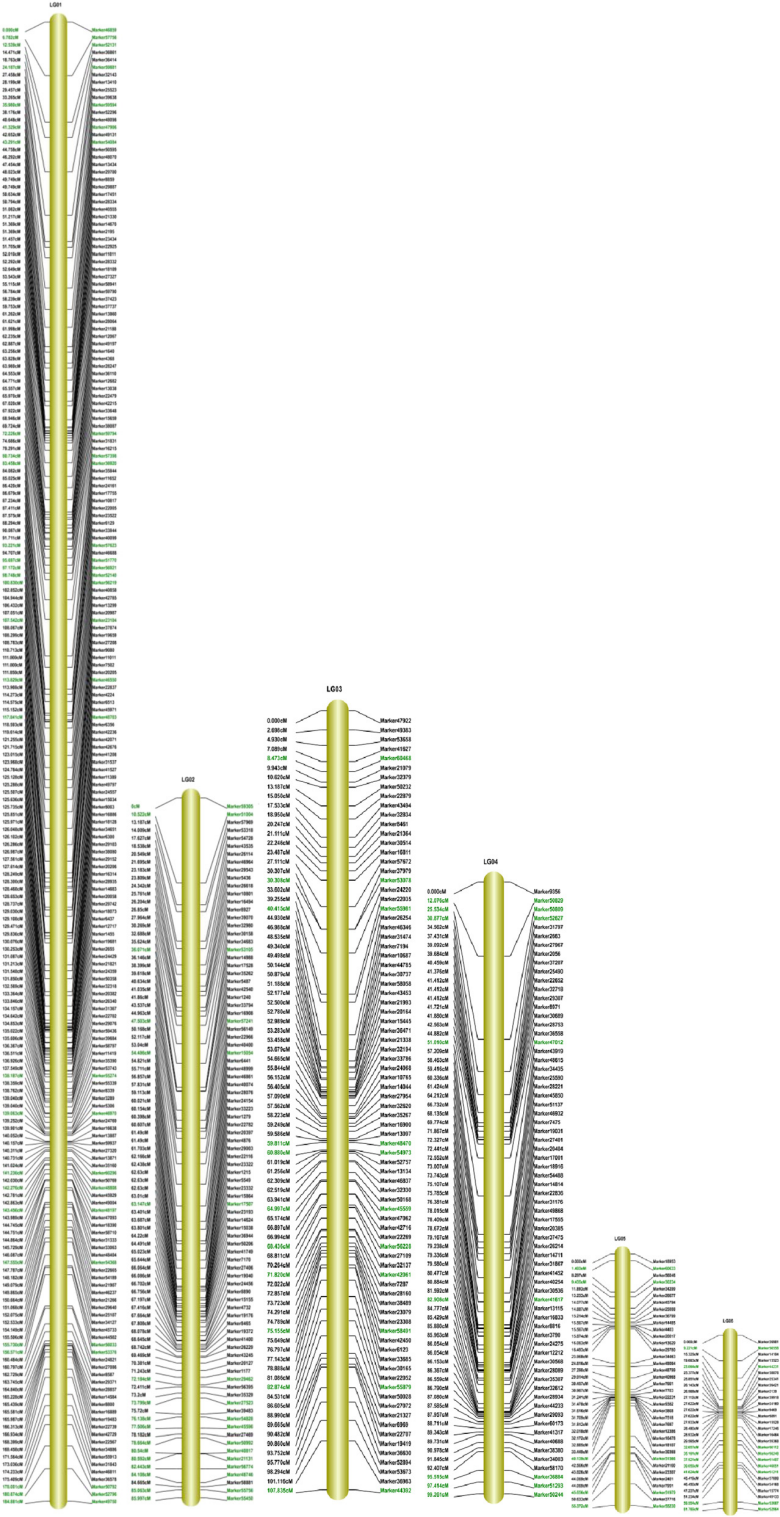
**Linkage group 1 to 6 of high-density linkage map for sesame.** Segregation distortion markers on the map are highlighted in green.

**Figure 4:**
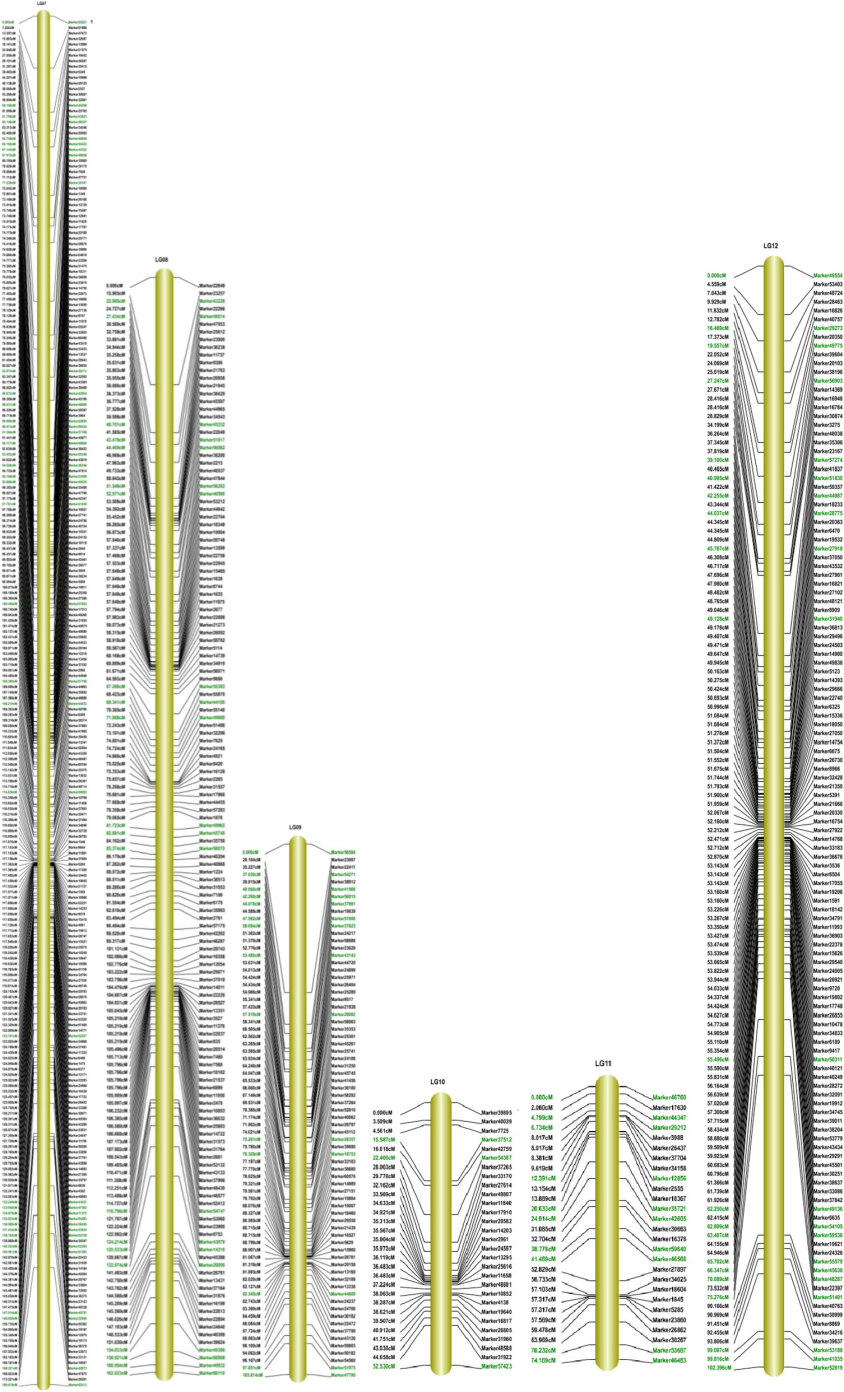
**Linkage group 7 to 12 of high-density linkage map for sesame.** Segregation distortion markers on the map are highlighted in green.

**Figure 5:**
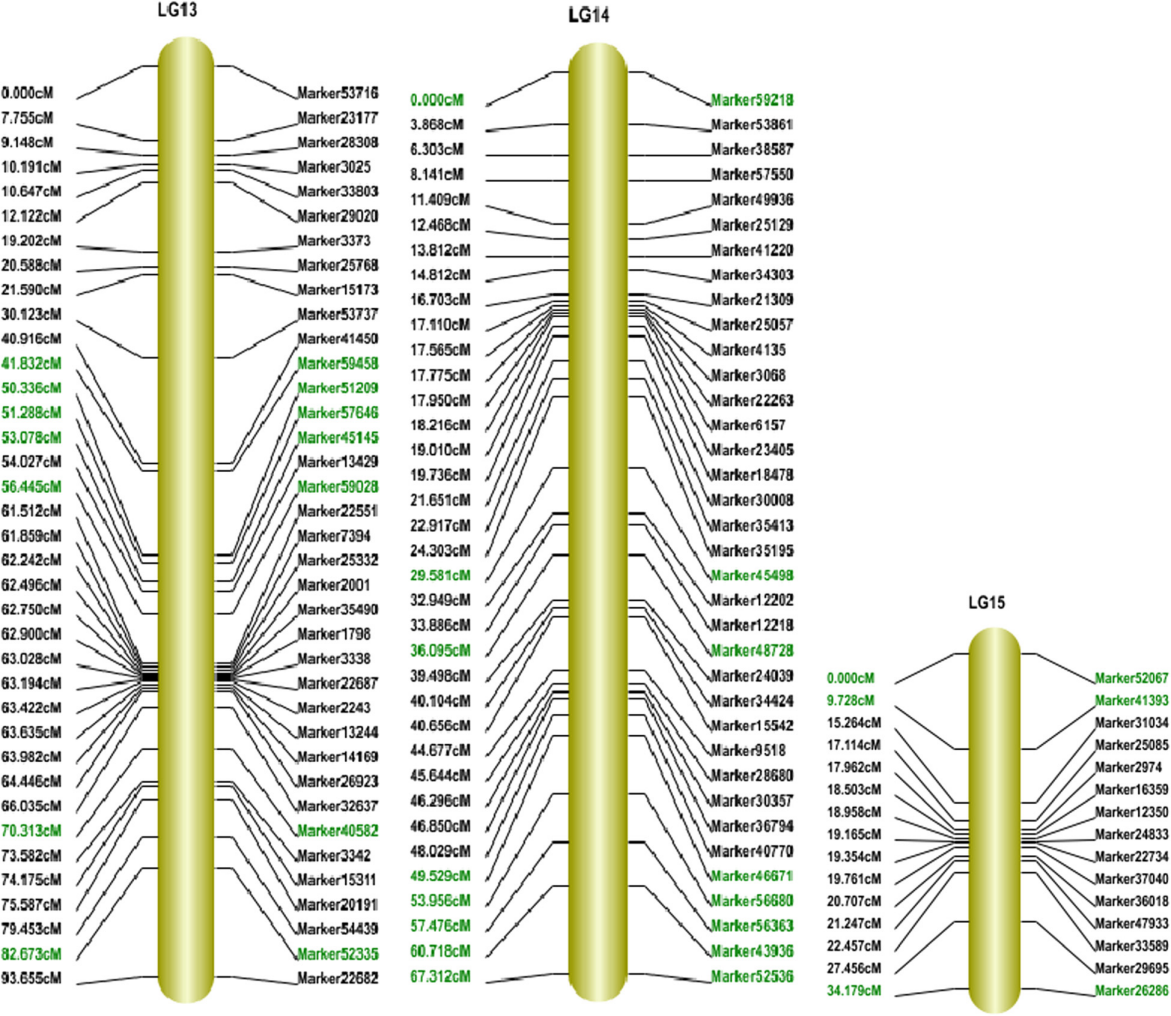
**Linkage group 13 to 15 of high-density linkage map for sesame.** Segregation distortion markers on the map are highlighted in green.

The final map included 1,233 markers on the 15 linkage groups (Figures 3, 4 and 5) and was 1,474.87 cM in length with an average distance of 1.20 cM between adjacent markers. To our knowledge, this map is the densest genetic linkage map to date for sesame. As shown in Table 2, the largest linkage group (LG) was LG7 with 247 markers, a length of 189.68 cM, and an average distance of only 0.77 cM between adjacent markers. The smallest LG was LG15, with only 15 markers, a length of 34.18 cM, and an average distance of 2.44 cM between adjacent markers. The degree of linkage between markers was reflected by 'Gap < = 5’ ranging between 78.57% and 98.85% with an average value of 91.76%. The largest gap on this map was 29.10 cM located in LG9.

**Table 2:** Description on basic characteristics of the 15 linkage groups

**Linkage group**	**Number of markers**				**Size (cM)**	**Average distance between markers (cM)**	**Largest gap (cM)**	**Gaps < =5**
	**Total**	**SNP_only**	**InDel_only**	**SNP&InDel**				
1	198	174	14	10	184.88	0.94	6.78	98.48%
2	88	78	8	2	86.00	0.99	10.52	98.85%
3	83	74	3	6	107.83	1.32	6.72	97.56%
4	69	58	7	4	99.26	1.46	13.46	92.65%
5	37	34	1	2	55.37	1.54	7.48	91.67%
6	28	26	2	0	61.77	2.29	9.22	88.89%
7	247	214	17	16	189.68	0.77	16.16	97.15%
8	142	124	11	7	162.02	1.15	15.91	97.87%
9	72	65	6	1	103.81	1.46	29.10	95.77%
10	29	21	2	6	52.53	1.88	11.02	85.71%
11	27	23	3	1	74.17	2.85	11.36	80.77%
12	125	112	6	7	102.40	0.83	14.89	97.58%
13	37	34	1	2	93.66	2.60	10.98	80.56%
14	36	33	2	1	67.31	1.92	6.59	94.29%
15	15	9	3	3	34.18	2.44	9.73	78.57%
Maximum	247	214	17	16	189.68	2.85	29.10	98.85%
Minimum	15	9	1	0	34.18	0.77	6.59	78.57%
Total	1233	1079	86	68	1474.87	/	/	/
Average	82.20	71.93	5.73	4.53	98.32	/	/	91.76%

'Gap < =5’ indicated the percentages of gaps in which the distance between adjacent markers was smaller than 5 cM.

### Distribution of markers types on the genetic map

**Figure 6:**
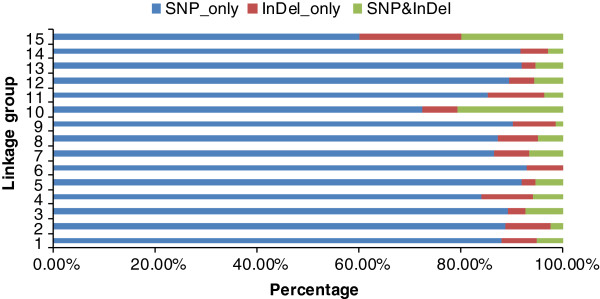
Percentages of diverse types of markers on each linkage group.

The genetic map had three types of markers including 1079 'SNP_only’ , 86 'InDel_only’ , and 68 'SNP&InDel’ markers. 'SNP_only’ was the predominant marker type accounting for 87.51% of the markers. Marker types in each of the 15 LGs were investigated (Table 2, Figure 6). LG15, the smallest LG, had the lowest percentage of 'SNP_only’ markers but had the highest percentage of 'InDel_only’ markers at 60% and 20%, respectively. LG10 had the highest percentage of 'SNP&InDel’ markers with 20.69%. The percentages of the three types of markers on the largest LG, LG7, were 86.64%, 6.88%, and 6.48%, respectively, which was very similar to the average distribution of marker types for all 15 LGs.

Among the 1,079 markers of the 'SNP_only’ type, 302 had two, or more than two, SNP loci, but the other 777 markers had a single SNP locus and accounted for 72% of the total. In total, 1,669 SNP loci were detected among the 1,233 markers on the final map and percentages of different SNP types were investigated (Table 3). Most of the SNPs were transition type SNPs with Y(T/C) and R(G/A) types accounting for 31.76% and 30.68%, respectively, of all SNP markers. The other four SNP types were transversions including S(G/C), M(A/C), K(G/T), and W(A/T) with percentages ranging from 8.69% to 10.96% and accounting for 37.56% of all SNPs.

**Table 3:** Statistic of mapped SNP marker types

**Type**	**Number**	**Ratio**
S(G/C)	145	8.69%
M(A/C)	148	8.87%
K(G/T)	151	9.05%
W(A/T)	183	10.96%
R(G/A)	512	30.68%
Y(T/C)	530	31.76%
Total	1669	100.00%

### Segregation distortion markers on the map

In total, 205 markers that showed significant (*P* < 0.05) segregation distortion were mapped onto the final map (Figure 3, 4 and 5) and were distributed mostly on the ends of LGs with linkage. The results of a χ^2^ test showed that segregation distortion markers were distributed on every LG with a distribution very similar to that of all markers (Table 4) except for LG6 and LG11. This means that one LG had a greater number of markers and segregation distortion markers at the same time. For example, the largest LG (LG7) had the highest percentage of segregation distortion markers (19.02%) and the smallest LG (LG15) had the lowest percentage of segregation distortion markers (1.46%). However, the frequency of segregation distortion markers on LG6 and LG11 was much higher than for other LGs at 32.14% and 37.04%, respectively. While 18 segregation distortion regions were detected on 11 LGs, LG1 and LG7 both had the largest number of segregation distortion regions.

**Table 4:** Distribution of segregation distortion markers

**Linkage group**	**All marker**		**Segregation distortion marker**		***χ***^***2***^	***P***	**Frequency of segregation distortion marker**	**SDR number**
	**Number**	**Percentage**	**Number**	**Percentage**				
1	198	16.06%	29	14.15%	0.524	0.469	14.65%	3
2	88	7.14%	17	8.29%	0.526	0.468	19.32%	1
3	83	6.73%	11	5.37%	0.841	0.359	13.25%	0
4	69	5.60%	8	3.90%	1.599	0.206	11.59%	2
5	37	3.00%	5	2.44%	0.222	0.638	13.51%	0
6	28	2.27%	9	4.39%	5.976	0.015	32.14%	1
7	247	20.03%	39	19.02%	0.122	0.727	15.79%	3
8	142	11.52%	21	10.24%	0.599	0.439	14.79%	1
9	72	5.84%	14	6.83%	0.25	0.617	19.44%	1
10	29	2.35%	3	1.46%	0.301	0.583	10.34%	0
11	27	2.19%	10	4.88%	8.664	0.003	37.04%	0
12	125	10.14%	21	10.24%	0.014	0.907	16.80%	2
13	37	3.00%	7	3.41%	0.121	0.728	18.92%	2
14	36	2.92%	8	3.90%	0.574	0.449	22.22%	1
15	15	1.22%	3	1.46%	0.445	0.505	20.00%	1
Total	1233		205				16.63%	18

*χ*^*2*^ and *P* indicate *χ*^*2*^ values with one degree of freedom and the corresponding probability, respectively.

SDR means segregation distortion region.

**Figure 7:**
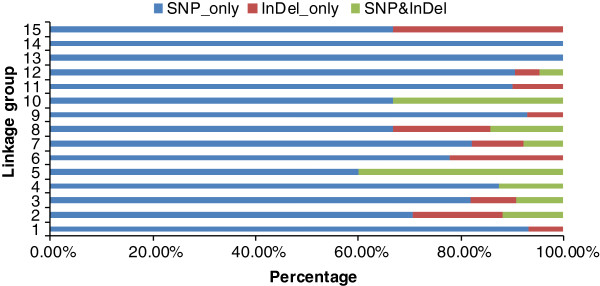
Percentages of diverse types of segregation distortion markers on each linkage group.

The 205 segregation distortion markers were of three types including 171 'SNP_only’-type markers, 20 'Indel_only’-type markers, and 14 'SNP&Indel’ markers with percentages of 83.41%, 9.76%, and 6.83%, respectively. This distribution was similar to the percentages observed for all markers of the three types, but the distributions of the three types of segregation distortion markers were different for each LG (Figure 7). Segregation distortion markers on LG13 and LG14 were all 'SNP_only’-type markers and only two types of segregation distortion markers were observed on eight other LGs. 'SNP_only’ and 'Indel_only’ segregation distortion markers were located on LG1, LG6, LG9, LG11, and LG15 and 'SNP_only’ and 'SNP&Indel’ segregation distortion markers were located in LG4, LG5, and LG10.

## Discussion

### The need to develop markers for sesame by sequencing

According to published results of research on sesame, the genetic diversity is low between germplasm resources [20-22,24,26,27] and genetic differences between cultivars are limited [23,25,30,32]. The close genetic relatedness between accessions could account for the low polymorphism of molecular markers. The polymorphism rates have been reported for a variety of markers in sesame including 20% for EST-SSR [28], 7.50% for EST-SSR, 9.00% for AFLP, 2.30% for RSAMPL [2], 11.59% for genic-SSR [30], 3.81% for cDNA-SSR [31], and 5.12% for SLAF markers (SNP and InDel in this study). The limited quantity of available markers and their low polymorphism rate makes construction of a genetic linkage map with high-density markers for sesame almost impossible using conventional methods. Genotyping by sequencing is a high-throughput technique that opens the door for the efficient development of large numbers of markers in a short time to generate enough polymorphic markers for high-density genetic map construction.

### The advantages of SLAF sequencing for developing markers

In contrast to inefficient, expensive, and time-consuming conventional methods of developing markers, bioinformatics analyses were carried out before SLAF sequencing; the reference genome of sesame (unpublished data) was analyzed, considering the information on genomic GC content, repeat conditions, and genetic characteristics. We designed an approach to ensure density, uniformity, and efficiency of marker development. The SLAF-seq method provided significant advantages such as the development of large numbers of markers having high accuracy with less sequencing, making it especially suited for analysis of species with low polymorphism like sesame. This study provides the first development of markers on large scale for sesame in total, 71,793 SLAF markers were developed based on high-throughput sequencing, and 1,272 polymorphic markers were identified for genetic linkage map construction. Marker integrity and accuracy were high and marker quality and quantity met the requirements for construction of a genetic map. Results also accurately reflect the genetic and polymorphism characteristics of sesame, which added to our understanding of the genetics of this species.

### Use of segregation distortion markers increased the genome coverage of the map

Because of the limited quantity of available molecular markers for sesame and their low polymorphism rate, additional markers such as segregation distortion markers should be used to construct a high-density genetic map. Segregation distortion is a common phenomenon that has been observed in many studies. The genotypic frequency of a segregation distortion marker deviates from a typical Mendelian ratio. This deviation may be caused by gametic selection [33], zygotic selection [34], or both. In this study, a genetic map was constructed first using Mendelian markers. Segregation distortion markers were then inserted in the existing map and the recombination fractions between markers were reestimated similar to the approach used by Wang et al. [35]. As a result, 205 markers (16.63%) were mapped onto the final map with a distribution on every LG, similar to the distribution of all markers. In addition to increasing the quantity of markers on the map, the genome coverage of the map increased. The presence of segregation distortion markers will not affect the use of this map for applications such as QTL mapping. Xu [36] showed that distorted markers can be used for QTL mapping with no detrimental effect on the results and can be beneficial if used properly. Zhang et al. [37] showed that segregation distortion could result in higher genetic variance than non-distortion and help to improve the detection of linked QTLs because distortion markers do not have a large effect on the position or effect estimations of QTL analysis. A new method of QTL mapping that uses markers for segregation distortion was developed by Xu and Hu [38].

### Future applications for the genetic map

To our knowledge, the genetic map presented in this paper is the densest map to date for sesame, though it is still not saturated, because it has 15 LGs, not 13 LGs as was expected. The map spans 1,474.87 cM with an average number of 82.20 markers per LG with an average distance of 1.20 cM between adjacent markers. The average distance in this map is much less than the 4.25 cM previously reported by Wei et al. [2]. More importantly, 93.03% of the markers on this genetic map are SNPs, the most abundant type of genetic variation between individuals. SNPs are sequence tagged markers with codominant inheritance so they are suitable for comparative genomic studies [39] and association mapping [40,41]. The results of this study not only provide mass markers for sesame, but also provide data useful for gene/QTL fine mapping, map-based gene isolation, and molecular breeding. Because these high-density linkage groups were constructed based on molecular markers developed at the whole genome level, they will serve as a reference for positioning sequence scaffolds on the physical map to assist in the assembly process of the sesame genome sequence.

## Conclusions

We report the first high-density genetic map for sesame. The map was constructed using an F_2_ population and was based on polymorphic markers developed using the SLAF-seq approach, which allowed the efficient development of a large number of markers in a short time. The results of this study will not only provide a platform for gene/QTL fine mapping, map-based gene isolation, and molecular breeding for sesame, but will also provide a reference to help position sequence scaffolds on the physical map and assist in the process of assembling the sesame genome sequence.

## Methods

### Plant material and DNA extraction

The F_2_ mapping population consisted of 107 individuals from a cross of 'Zhongzhi No. 13’ (female parent) and 'Shandong Jiaxiang Sesame’ (male parent) grown at the Oil Crops Research Institute of the Chinese Academy of Agricultural Sciences in Wuhan. Young healthy leaves from the two parents and F_2_ individuals were collected, frozen in liquid nitrogen, and used for DNA extraction. Total genomic DNA was prepared from each plant according to the cetyltrimethylammonium bromide (CTAB) method [42] with some modification to the components of the CTAB buffer (8.18 g sodium chloride and 2 g CTAB in a total volume of 100 ml of 20 mM EDTA, 100 mM Tris, pH 8.0). DNA concentration and quality were estimated with an ND-1000 spectrophotometer (NanoDrop, Wilmington, DE, USA) and by electrophoresis in 0.8% agarose gels with a lambda DNA standard.

### SLAF library construction and high–throughput sequencing

The procedure was performed as described by Sun et al. [19] with small modifications. Briefly, a pilot SLAF experiment was performed to establish conditions to optimize SLAF yield, avoid repetitive SLAFs, and obtain an even distribution of SLAFs for maximum SLAF-seq efficiency. Based on the result of the pilot experiment, the SLAF library was constructed as following. Genomic DNA was first incubated at 37°C with *Mse*I [New England Biolabs (NEB), Ipswich, MA, USA], T4 DNA ligase (NEB), ATP (NEB), and *Mse*I adapter. Restriction/ligation reactions were heat-inactivated at 65°C and digested with *EcoR*I and *Bfa*I restriction enzymes at 37°C. Then, polymerase chain reactions (PCR) were carried out in the reaction solutions containing the diluted restriction/ligation samples, dNTP, Taq DNA polymerase (NEB), and *Mse*I-primer containing barcode 1. PCR products were purified using an E.Z.N.A.® Cycle Pure Kit (Omega Bio-Tek, Norcross, GA, USA) and pooled. Pooled samples were incubated at 37°C with *Mse*I, T4 DNA ligase, ATP, and Solexa adapter, purified using a Quick Spin column (Qiagen, Hilden, Germany), and run on a 2% agarose gel. Fragments of 400–450 bp (with indices and adaptors) were isolated using a Gel Extraction Kit (Qiagen) and subjected to PCR amplification with Phusion Master Mix (NEB) and Solexa Amplification primer mix (Illumina, Inc., San Diego, CA, USA) to add barcode 2 according to the Illumina sample preparation guide. After samples were gel purified, DNA fragments (SLAFs) of 400–450 bp were excised and diluted for pair-end sequencing on an Illumina High-seq 2000 sequencing platform (Illumina, Inc; San Diego, CA, U.S.) at Biomarker Technologies Corporation in Beijing (http://www.biomarker.com.cn/english/). Real-time monitoring was performed for each cycle during sequencing, the ratio of high quality reads with quality scores greater than Q20 (means a quality score of 20, indicating a 1% chance of an error, and thus 99% confidence) in the raw reads and guanine-cytosine (GC) content were calculated for quality control.

### SLAF-seq data grouping and genotype definition

All SLAF pair-end reads with clear index information were clustered based on sequence similarity as detected by BLAT [43] (-tileSize = 10 -stepSize = 5). Sequences with over 90% identity were grouped in one SLAF locus as described by Sun et al. [19]. Alleles were defined in each SLAF using the minor allele frequency (MAF) evaluation. Because sesame is a diploid species, one locus contains at most four SLAF tags, so groups containing more than four tags were filtered out as repetitive SLAFs. In this study, SLAFs with a sequence depth of less than 107 were defined as low-depth SLAFs and were filtered out. SLAFs with 2, 3, or 4 tags were identified as polymorphic SLAFs and considered to be potential markers. Polymorphic markers were classified into eight segregation patterns (ab × cd, ef × eg, hk × hk, lm × ll, nn × np, aa × bb, ab × cc and cc × ab). An F_2_ population is obtained by selfing the F1 of a cross between two fully homozygous parents with genotype aa or bb. Therefore, the study only used the SLAF markers which segregation patterns were aa × bb for genetic map construction. The average sequence depths of SLAF markers were greater than 20-fold in parents and greater than 14-fold in progeny. Any a progeny contained more than 80% of the SLAF markers in the parents, ie, 80% integrity of SLAF markers in individuals.

### Segregation distortion analysis and genetic map construction

Marker segregation ratios were calculated using the chi-square test. Markers showing significant (*P* < 0.05) segregation distortion were initially excluded from the map construction and were then added later as accessory markers. A region on the map with more than three adjacent loci that showed significant (*P* < 0.05) segregation distortion was defined as a segregation distortion region (SDR) [44]. The recombination rates between markers were calculated using JoinMap 4.0 software [45] and the genetic map was constructed using a logarithm of odds (LOD) threshold ≥4.0 and a maximum recombination fraction of 0.4. Map distances in centi-Morgans were calculated using the Kosambi mapping function [46].

## Competing interests

The authors declare that they have no competing interests.

## Authors’ contributions

XZ, YZ and LW designed and organized the entire project. LW, DL and XD performed the experiments. YZ, HX, CM and WH analyzed the data. YZ, HX and XZ drafted the manuscript. All authors read and approved the final manuscript.

## Supplementary Material

Additional file 1: Table S1 Details of primer sequences. Click here for file

Additional file 2: Table S2 Details of primer genotypes for F_2_ individuals. Click here for file
